# Mast cells: a double-edged sword in inflammation and fibrosis

**DOI:** 10.3389/fcell.2024.1466491

**Published:** 2024-09-17

**Authors:** Xufang Wang, Peipei Zhang, Yuxin Tang, Yanlin Chen, Enchao Zhou, Kun Gao

**Affiliations:** ^1^ Jiangsu Province Key Laboratory of Tonifying Kidney and Anti-senescence, Department of Nephrology, Jiangsu Province Hospital of Chinese Medicine, Affiliated Hospital of Nanjing University of Chinese Medicine, Nanjing, China; ^2^ The First Affiliated Hospital of Zhejiang Chinese Medical University (Zhejiang Provincial Hospital of Chinese Medicine), Hangzhou, China

**Keywords:** mast cell, inflammation, fibrosis, fibroblast, innate immunity, adaptive immunity

## Abstract

As one of the key components of the immune system, mast cells are well known for their role in allergic reactions. However, they are also involved in inflammatory and fibrotic processes. Mast cells participate in all the stages of acute inflammatory responses, playing an immunomodulatory role in both innate and adaptive immunity. Mast cell-derived histamine, TNF-α, and IL-6 contribute to the inflammatory processes, while IL-10 mediates the suppression of inflammation. Crosstalk between mast cells and other immune cells is also involved in the development of inflammation. The cell–cell adhesion of mast cells and fibroblasts is crucial for fibrosis. Mast cell mediators, including cytokines and proteases, play contradictory roles in the fibrotic process. Here, we review the double-edged role of mast cells in inflammation and fibrosis.

## 1 Introduction

Mast cells (MCs) are important components of the innate and adaptive immune system (Tikoo et al., 2018). MCs are found in almost all kinds of tissues and organs, especially at sites close to the external environments, such as mucosal membranes, the skin, and perivascular areas, acting as sentinels (Piliponsky and Romani, 2018). The role of MCs has been widely investigated in allergic diseases such as urticaria, asthma, and rhinitis. It has been reported that the mediators released by activated MCs are crucial in allergic diseases (Anastasia et al., 2024). A protective role of mast cells in allergic responses has been reported recently (Thomas et al., 2023; Esther et al., 2023). These studies show the biphasic role of MCs in allergy. Because MCs are also demonstrated to be involved in inflammation and fibrosis (Ribatti et al., 2020; Guo et al., 2023), we will discuss the activating and inhibitory effects of MCs in these processes.

Using single-cell transcriptomics analysis, scientists proved that MCs derived from CD34^+^ pluripotent progenitor cells reside in the bone marrow (Wu et al., 2022). Mast cell progenitors leave the bone marrow and migrate into peripheral tissue, where they finish the process of maturation (Tahereh et al., 2022). Stem cell factor (SCF), which is the ligand of CD117, plays an important role in almost every stage of MC differentiation (Terhorst-Molawi et al., 2023). Mature MCs express a variety of stimulatory and inhibitory receptors on the cell surface (Daniel et al., 2019). Once activated through stimulatory receptors, MCs might release granules that contain a variety of mediators, including preformed mediators, newly synthesized lipid mediators, cytokines, and chemokines (Petri and Ilze, 2017). Due to the diversity of mediators in different MC subtypes, human mast cells are divided into two major subtypes: mast cells whose granules contain mostly tryptase (MC_T) and mast cells whose granules contain tryptase and chymase (MC_TC) (Baba et al., 2017). However, an intermediate phenotype linked to both MC_T and MC_TC was observed in human airway tissues (Dwyer et al., 2021). Another study identified six mast cell clusters in different human tissues (Tauber et al., 2023). These studies showed the natural diversity and heterogeneity of mast cells.

## 2 Mast cells in acute inflammatory responses

### 2.1 Mast cells in innate immunity

MCs are involved in innate inflammatory responses such as bacterial infection, venom damage, and tissue injury (Chompunud Na Ayudhya et al., 2020). MCs express a variety of stimulatory receptors that function in innate immunity (Daniel et al., 2019). Agonists of toll-like receptors and Mas-related G protein-coupled receptors (MRGPR), complement anaphylatoxins C3a and C5a, and endogenous peptides are involved in mast cell activation (Cho et al., 2021; Roy et al., 2021; Laumonnier et al., 2023). Unlike the adaptive signals, the innate stimuli lead to the release of small and spherical granules rapidly through the activation of MCs. Those small granules induce local and transient inflammation in innate inflammatory responses (Gaudenzio et al., 2016). Previous studies showed that the activation of MRGPRX2 enhanced the clearance of *Staphylococcus aureus* from infected mouse skin (Arifuzzaman et al., 2019). MCs were also demonstrated to protect against snake and honeybee toxins by releasing proteases (Yuki et al., 2023). In addition, substance P (SP) could activate Mrgprb2 and mediate thermal hyperalgesia and non-histaminergic itch (Green et al., 2019; Meixiong et al., 2019). These studies revealed the proinflammatory role of MCs in innate immunity.

**FIGURE 1:**
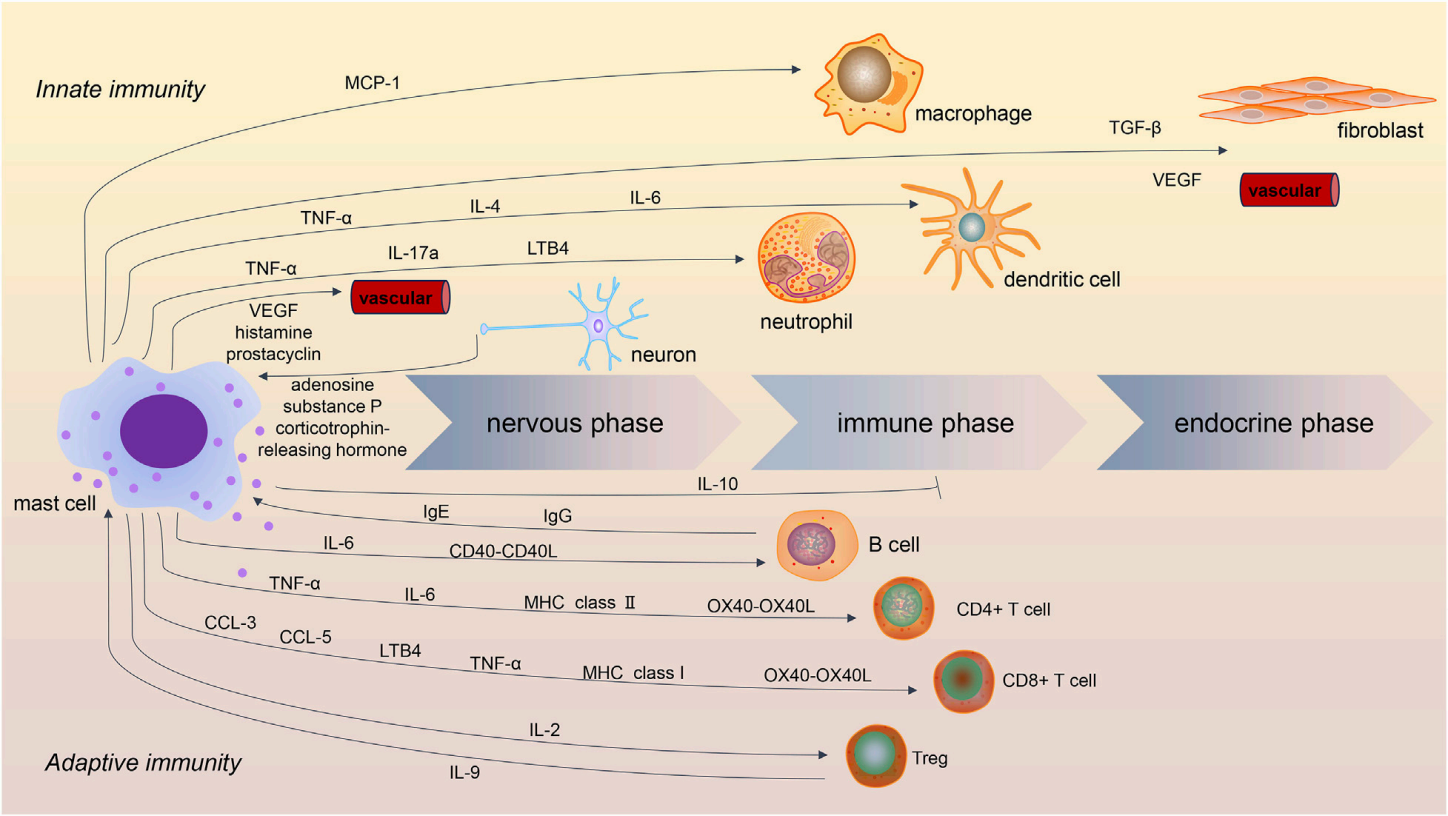
Role of mast cells in acute inflammatory processes. Mast cells are involved in innate and adaptive immunity in the three stages of acute inflammation. In addition to IL-10, most mast cell mediators are proinflammatory. Crosstalk between mast cells and macrophages, dendritic cells, and neutrophils contributes to innate immunity, while the interaction of mast cells with B cells and T cells contributes to adaptive immunity. Crosstalk between mast cells and Treg has been shown to be immunosuppressive in inflammatory processes.

A series of three successive phases had been described in the acute inflammatory responses (Aller et al., 2004). MCs were shown to be involved in all three stages (Figure 1) (Aller et al., 2019). The first phase of acute inflammation is the nervous phase, which manifests as vascular permeability changes through sensory and motor alterations. The activation of the hypothalamic–pituitary–adrenal axes and sympathetic nervous system was observed in acute tissue injury. In the immediate phase of inflammation, crosstalk of MCs and sensory nerves was crucial (Toyoshima and Okayama, 2022). Activation of the neuroendocrine system induces the release of activating signals, including adenosine, corticotrophin-releasing hormone, and substance P. Those stimuli could activate MCs. Activated MCs then release mediators such as histamine, vascular endothelial growth factor (VEGF), and prostacyclin, which increase vascular permeability and smooth muscle contraction. In addition, histamine, tryptase, and nerve growth factors released by MCs could stimulate nerve fibers and amplify the inflammatory courses.

In the later phase of inflammation, also called the immune phase, cytokines released by MCs contribute to the recruitment and maturation of other immune cells, such as macrophages, dendritic cells, and neutrophils. In this stage, MCs mainly play immunoregulatory roles in innate and adaptive immunity. Focusing on innate inflammation, MCs have been shown to contribute to macrophage recruitment and polarization by releasing cytokines such as monocyte chemotactic protein-1 (MCP-1) (Elieh Ali Komi et al., 2020; Zhang et al., 2020). MCs can also modulate neutrophils and dendritic cells. In the process of tissue injury, perivascular MCs were demonstrated to induce neutrophil recruitment and activation through the release of tumor necrosis factor (TNF)-α and interleukin (IL)-17a (Jan et al., 2021; Katia et al., 2013; Régis et al., 2022). Moreover, during bacterial infection, MC-derived leukotriene B4 (LTB4) contributed to host defense by mediating early neutrophil influx and bacterial clearance (Van Bruggen et al., 2023). Similarly, MC-derived TNF-α and IL-6 were shown to promote the migration and maturation of dendritic cells (Jan et al., 2015; Dudeck et al., 2019). Furthermore, MC-derived IL-4 mediated dendritic cell activation at the site of inflammation (Friedel et al., 2024). These results show that mast cells, macrophages, neutrophils, and dendritic cells comprise an important barrier of innate immunity.

Finally, in the last phase of acute inflammation, referred to as the endocrine phase, MCs could influence the microenvironment by inducing fibrogenesis and angiogenesis. MC-derived transforming growth factor (TGF)-β, VEGF, and fibroblast growth factor (FGF) are involved in this stage (Kyritsi et al., 2021). Those mediators are also essential in chronic inflammation and fibrosis.

### 2.2 Mast cells in adaptive immunity

MCs are believed to be a critical bridge in the transition from innate to adaptive immunity (Cardamone et al., 2016). MCs express high-affinity immunoglobulin E (IgE) receptors and several low-affinity IgG receptors on the cell surface (Daniel et al., 2019). These receptors allow antibodies to enroll MCs into immune responses in the presence of antigens. The role of MCs has been widely investigated in IgE-induced anaphylaxis (Dispenza et al., 2023). IgE and the high-affinity IgE receptors on MCs were also shown to be crucial for acquired resistance to honeybee venom (Marichal et al., 2013). In addition, the crosstalk between MCs and adaptive immune cells, including B cells and T cells, was essential for adaptive immunity.

MCs have been found to induce the activation and differentiation of B cells both in mouse models and humans (Breitling et al., 2017; Rurik et al., 2021). Direct cell–cell contact was reported between MCs and B cells. The CD40-CD40L interaction play- a key role in MC-mediated B-cell activation (Gwan Ui et al., 2015; Viviana et al., 2020). In addition, MC-derived IL-6 was involved in B cell differentiation and immunoglobulin A (IgA) secretion (Merluzzi et al., 2010; Valeri et al., 2021). Because B-cell-derived IgE could re-activate MCs and result in more mediator release, MC-B-cell interaction might exaggerate the adaptive inflammatory responses.

Human MCs were shown to release chemokines such as chemokine (C-C motif) ligand (CCL)3 and CCL5 to recruit cytotoxic cells, including CD8+T cells, during virus infection (Yana et al., 2021; Chen et al., 2023). MC-derived LTB4 and TNF-α were crucial for the amplifying of CD8^+^ T cells dominated adaptive immunity (Jan et al., 2015; Bodduluri et al., 2018). MCs were shown to be capable of antigen presentation via major histocompatibility complex (MHC) class I molecules to CD8+T cells (R et al., 1996). This crosstalk proved to be important in regulating CD8+T-cell proliferation, cytokine secretion, and cytotoxic activity. MCs were demonstrated to express costimulatory molecules OX40L for T-cell activation in human skin (Marta et al., 2014). Similarly, MCs are involved in the recruitment, activation, and differentiation of CD4^+^ T cells. Most of the modulating function of MCs on CD4^+^ T cells depends on the expression of MHC class Ⅱ molecules and OX40L (Sahar et al., 2017). TNF-α and IL-6 are also crucial in establishing synaptic contacts (Nicolas et al., 2013). Interestingly, evidence showed that MCs treated with nicotinamide adenine dinucleotide (NAD)+ could mediate CD4^+^ T-cell differentiation independently of MHC II and T-cell receptor signaling machinery (Hector et al., 2018).

In addition to the roles of initiator and effector in innate immunity, MCs are also important in the host’s transition from innate to adaptive immunity. The crosstalk between MCs and other immune cells indicated the immunomodulatory ability of MCs. The immunomodulatory ability might be more important than the effector-cell function under given circumstances. Further exploration is needed to understand how to regulate MCs in inflammatory processes.

### 2.3 Role of mast cells in the suppression of inflammatory responses

MCs were demonstrated to be suppressors of inflammatory responses. The anti-inflammatory role of MCs is partially dependent on the release of IL-10. Using three different types of MC-deficient mice and mice with ablated MC-derived IL-10, researchers showed that MC-derived IL-10 could limit inflammation and tissue pathology in contact hypersensitivity (Reber et al., 2017). In another study using IL-33 for the activation of synovial MCs, researchers found that the production of IL-10 and histamine was elevated, resulting in the suppression of monocyte-mediated disease activity in rheumatoid arthritis (Rivellese et al., 2015). MC-derived IL-10 also functions in graft-versus-host disease (GVHD). MC-derived IL-10 significantly reduced GVHD by decreasing conventional T-cell proliferation. This immunosuppressive ability was independent of Tregs (Leveson-Gower et al., 2013). In experimental murine myeloperoxidase (MPO)-ANCA-associated vasculitis (AAV), MC-derived IL-10 enhanced the immunosuppressive function of Treg and played a protective role in MPO-related inflammation (Gan et al., 2012; Gan et al., 2016).

In addition to IL-10, the immunosuppressive function of MCs also depends on the crosstalk of MCs and regulatory T cells. IL-9 produced by Treg is important in MC recruitment and activation, which then mediate regional immune suppression in tolerant allografts. MC-deficient mice are not capable of establishing long-term allograft tolerance (Li-Fan et al., 2006). In addition, MC-derived IL-2 is involved in suppressing chronic allergic dermatitis by promoting Treg expansion (Keith et al., 2023). MCs are also crucial for the resistance of streptozotocin-induced type 1 diabetes mellitus by promoting Treg-mediated immunological tolerance (Carlos et al., 2015).

Although many studies have discussed the proinflammatory and anti-inflammatory roles of MCs, it is still unclear which signals mediate the immunosuppressive function of MCs. One study showed that the MC stabilizer disodium cromoglycate inhibited MC degranulation without affecting IL-10 production and resulted in the protection of AAV disease activity (Gan et al., 2016). This study suggested that some medications might limit the deleterious function and retain the protective ability of MCs. Perhaps there will be more targeted treatments that regulate the function of MCs in inflammatory processes in the future.

## 3 Mast cells and fibrosis

### 3.1 Mast cells in the activation of fibroblasts

**FIGURE 2:**
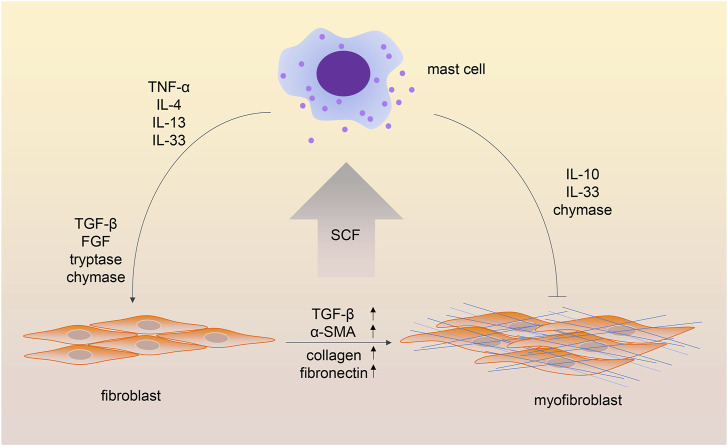
Crosstalk between mast cells and fibroblasts in fibrotic processes. The cell–cell adhesion between mast cells and fibroblasts depends on the stem cell factor-Kit (SCF-KIT) interaction. In addition, mast cell-derived cytokines and chemokines contribute to the transition of fibroblasts into myofibroblasts. Interestingly, some mast cell mediators have been shown to have both profibrotic and antifibrotic functions.

The transition of fibroblasts into myofibroblasts was shown to induce excessive deposition of extracellular matrix and then cause fibrosis in different tissues and organs (Younesi et al., 2024). Crosstalk between MCs and fibroblasts has been investigated in recent decades (Figure 2). One study showed that co-culture of dermal fibroblasts with human MC significantly enhanced contraction of the three-dimensional collagen lattices; the addition of antibodies against stem cell factor (SCF) and c-kit resulted in the inhibition of gel contraction. Nevertheless, adding the supernatants of MCs to cultured fibroblasts did not enhance the speed of gel contraction, which indicates the importance of cell–cell contact (Yamamoto et al., 2000). Several studies showed cell adhesion between MCs and fibroblasts (Yaksh et al., 2019; Pincha et al., 2018). Murine MCs adhere to fibroblasts through SCF-KIT interaction (Adachi et al., 1992), while the adhesion of human MCs and fibroblasts depends on the expression of the cell adhesion molecule (CADM)1-kit mechanism (Moiseeva et al., 2013). This cell–cell adhesion is the foundation of contact-dependent cell communication.

In addition to the contact-dependent cell communication, mediators released by both cells are crucial in the MC-fibroblast crosstalk. It is believed that fibroblasts triggered MC activation through the production of SCF (Luo et al., 2022). Meanwhile, several MC-derived mediators, such as TGF-β, are involved in activating fibroblasts. TNF-α was shown to be crucial for the MC-fibroblast interaction in myocardial fibrosis (Zhang et al., 2011). MC-derived IL-13/IL-4 is involved in disease progression in myelofibrosis (Melo-Cardenas et al., 2022). MCs could release IL-33, which stimulates fibroblasts and enhances collagen expression (Wulff et al., 2019). MC tryptase was demonstrated to induce lung fibroblast migration via protease-activated receptor-2 (PAR-2) activation (Bagher et al., 2018). In addition, MC tryptase was shown to promote inflammatory bowel disease-induced intestinal fibrosis through the PAR-2/Akt/mammalian target of the rapamycin (mTOR) pathway of fibroblasts (Liu et al., 2021). MC chymase was found to promote fibroblast proliferation and collagen synthesis through the activation of the TGF-β1/Smads signaling pathway (Chen et al., 2017). Chymase was also shown to be a major source of angiotensin II (Ang II) production (Ahmad and Ferrario, 2018), and Ang II was widely investigated for its pro-fibrosis role in different diseases (Lv et al., 2021).

### 3.2 Mast cells in the inhibition of fibrosis

An antifibrotic role of MCs was also observed in several studies. One study observed the difference between the unilateral ureteral obstruction (UUO) model in W/W^v^ mice and the UUO model in the wild type. Higher levels of renal interstitium fibrosis, more infiltrating immune cells, and higher tissue levels of TGF-β1 were observed in MC-deficient mice, which indicated the protective role of MCs in renal fibrosis. A possible explanation of the protective role of MCs was that MC-derived heparin could inhibit TGF-β1 production (Kim et al., 2009; Beghdadi et al., 2013). As mentioned above, MCs could produce anti-inflammatory mediators, including IL-10 and IL-33 (Stephen et al., 2020). IL-10 has been shown to attenuate cardiac fibrosis by inhibiting TGF-β-induced myofibroblast differentiation (Verma et al., 2017). Another study observed that IL-33 impaired fibroblast migration and gel contraction in age-related macular degeneration (Theodoropoulou et al., 2015). Because IL-33 has been reported for its profibrotic role in diseased states, the function of IL-33 in fibrosis might be contradictory. Another MC mediator was proved to be both profibrotic and antifibrotic. MC chymase was shown to protect against renal fibrosis by activating matrix metalloproteinases and degrading interstitial deposits of fibronectin (Beghdadi et al., 2013; MacColl and Khalil, 2015).

### 3.3 Confounding factors of mast cells in fibrosis

Unlike the role of MCs in the inflammatory process, the role of MCs in fibrosis is more complicated. The anti-inflammatory function of MCs is mostly mediated by IL-10 and crosstalk between MCs and regular T cells. However, in fibrotic processes, both a profibrotic and antifibrotic role can be observed in a specific MC-derived mediator. In addition, results from cell culture and animal models are often contradictory. One explanation is that some of the MC-deficient animal models are not appropriate for the study under the given circumstances. Some reactions have been demonstrated to be induced by the other consequences of the genetic mutation instead of the absence of MCs (Chmelař et al., 2016). The different types of stimuli may also be responsible for the paradoxical results. The reactions of MCs are not consistent for acute tissue injury and chronic tissue damage. One study investigated the role of MCs in both the early and late phases in murine models of acute and chronic renal ischemia-reperfusion injury (IRI). MCs were found to promote fibrosis in the acute phase of IRI but not in the chronic phase of the disease (Danelli et al., 2017). Moreover, the most widely used MC stabilizers, disodium cromoglycate and ketotifen, can inhibit IgE-mediated MC degranulation but have no impact on mediators independently of degranulation. A high concentration of disodium cromoglycate has an effect beyond its inhibition on MCs (Oka et al., 2012). This makes targeting the antifibrotic effects of MCs more challenging.

## 4 Conclusion

The heterogeneity and diversity of mediators make the role of MCs contradictory in inflammation and fibrosis. For further research, animal models should be carefully selected to eliminate the effect of MCs. Using two or more MC-deficient animal models, as well as MC-engrafted methods, will be more precise than using one single animal model. In addition, most research animal models are designed for acute injury; perhaps an animal model of chronic damage may reflect the functions of MCs in chronic inflammation and fibrosis more exactly. Moreover, the specificity of MC stabilizers is not satisfactory; targeting a single mediator of MCs will be more important in research and clinical applications.
